# Ultra-wide range field-dependent measurements of the relaxivity of Gd_1−x_Eu_x_VO_4_ nanoparticle contrast agents using a mechanical sample-shuttling relaxometer

**DOI:** 10.1038/srep44770

**Published:** 2017-03-20

**Authors:** Ching-Yu Chou, Mouna Abdesselem, Cedric Bouzigues, Minglee Chu, Angelo Guiga, Tai-Huang Huang, Fabien Ferrage, Thierry Gacoin, Antigoni Alexandrou, Dimitris Sakellariou

**Affiliations:** 1 Departement de Chimie, Ecole Normale Superieure, PSL Research University, UPMC Univ Paris 06, CNRS, Laboratoire des Biomolecules (LBM), 24 rue Lhomond, 75005 Paris, France; 2 Sorbonne Universites, UPMC Univ Paris 06, Ecole Normale Superieure, CNRS, Laboratoire des Biomolecules (LBM), Paris, France; 3 NIMBE, CEA-CNRS, Université Paris-Saclay, CEA Saclay, 91191 Gif-sur-Yvette Cedex, France; 4 Laboratoire d’Optique et Biosciences, Ecole polytechnique, CNRS, INSERM, Université Paris-Saclay, 91128, Palaiseau, France; 5 Institute of Physics, Academia Sinica, Nankang, Taipei, 115, ROC, Taiwan; 6 Institute of Biomedical Science, Academia Sinica, Nankang, Taipei, 115, ROC, Taiwan; 7 Laboratoire de Physique de la Matière Condensée, Ecole polytechnique, CNRS, Université Paris-Saclay, 91128, Palaiseau, France.

## Abstract

The current trend for Magnetic Resonance Imaging points towards higher magnetic fields. Even though sensitivity and resolution are increased in stronger fields, T1 contrast is often reduced, and this represents a challenge for contrast agent design. Field-dependent measurements of relaxivity are thus important to characterize contrast agents. At present, the field-dependent curves of relaxivity are usually carried out in the field range of 0 T to 2 T, using fast field cycling relaxometers. Here, we employ a high-speed sample shuttling device to switch the magnetic fields experienced by the nuclei between virtually zero field, and the center of any commercial spectrometer. We apply this approach on rare-earth (mixed Gadolinium-Europium) vanadate nanoparticles, and obtain the dispersion curves from very low magnetic field up to 11.7 T. In contrast to the relaxivity profiles of Gd chelates, commonly used for clinical applications, which display a plateau and then a decrease for increasing magnetic fields, these nanoparticles provide maximum contrast enhancement for magnetic fields around 1–1.5 T. These field-dependent curves are fitted using the so-called Magnetic Particle (MP) model and the extracted parameters discussed as a function of particle size and composition. We finally comment on the new possibilities offered by this approach.

Spatial resolution, signal-to-noise and contrast-to-noise ratios are crucial quality factors of Magnetic Resonance Imaging (MRI). Even though clinical MRI systems are nowadays operating at 1.5 T or 3.0 T, the trend for research MRI scanners is towards increasing magnetic fields, such as 7.0 T, 9.4 T^1^, or more recently even 11.7 T^2^. The drive towards ultra-high magnetic fields is motivated by subsequent benefits in terms of ultra-high spatial resolution, signal-to-noise ratio and spectroscopic information.

One of the most common modality for clinical imaging is based on contrast due to variations of the longitudinal relaxation time *T*_1_. However, the extension of *T*_1_ contrast to high fields is not straightforward. Although the measured *T*_1_ in tissues and blood have been found to rise with increasing fields^3^,^4^, the difference in *T*_1_ for different tissues becomes less pronounced at higher magnetic fields (B_0_ > 2 T), leading to a reduction in imaging contrast. This points to the necessity for increasing the image contrast with appropriately designed contrast agents^5^,^6^. The T_1_ contrast agents currently used in clinical practice are based on Gadolinium (Gd^3+^) chelates which generate contrast by reducing the relaxation time of water protons ^1^H ref. 7. However, their efficiency as contrast agents strongly decreases above 0.2 T ref. 7.

One approach to increase imaging contrast at high magnetic field was to synthesize multinuclear Gd^3+^ complexes^8^ or Europium (Eu^2+^) complexes^9^. Other material design approaches have proposed paramagnetic nanoparticles as promising materials for contrast agents in high magnetic fields^10^. In particular, Gd_2_O_3_^11^, GdS^12^, GdPO_4_^13^ and GdVO_4_^14^ nanoparticles have been proposed as efficient *T*_1_ contrast agents. In several of these refs^11^,^12^,^13^, doping with luminescent rare-earth ions like Eu^3+^ added a fluorescence imaging functionality, and, in the case of GdVO_4_, doping with Eu^3+^ ions lead to multifunctional particles allowing MRI contrast enhancement together with both optical imaging and reactive oxygen species detection^14^.

The physical principle of MRI contrast agents is the perturbation of relaxation mechanisms in tissues or solutions by using the paramagnetic effects to differentially alter the relaxation times in the targeted tissues. Currently, clinical usage is dominated by gadolinium-based T_1_-contrast agents using Gd^3+^ ions embedded in chelate molecules, which increase imaging contrast on T_1_-weighted images, shorten acquisition time and improve diagnostic confidence^15^,^16^. The performance of contrast agents is characterized by the relaxivity defined as the change in relaxation rate (*R*_1_ = 1/*T*_1_, s^−1^) of water protons upon addition of the contrast agent, normalized to the concentration of the contrast agent [*CA*] in units of mM:


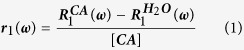


The higher the relaxivity, the higher the contrast of the image, which is, as indicated by the definition of the relaxivity, dependent upon the magnetic field B_0_ and the corresponding Larmor frequency *ω*. The fluctuations of the dipolar interactions between the nuclei of the solvent and the electron spin of the contrast agent, as well as the fluctuations of the electronic spin of the contrast agent are responsible for the nuclear spin relaxation of the solvent. Because of the nonlinear magnetic field dependence of this relaxation mechanism, the field-dependent curves of relaxivity ***r***_1_(***ω***), the so-called “Nuclear Magnetic Relaxation Dispersion” curves (NMRD), are also nonlinear.

In common NMRD studies, *T*_1_ relaxation is measured in a range of magnetic field strengths using pulsed magnets, commonly known as fast field cycling relaxometers. Their range of magnetic fields is limited from μT up to 2 T^15^,^16^,^17^ (~40 Hz–80 MHz for proton MRI), because higher fields would require higher electrical power and tremendous heat dissipation^17^. This technique does cover several decades in frequency, however the data in the literature usually stop at about ~60 MHz. Thus, relaxivity measurements at fields higher than 1.5 T are usually done at discrete magnetic field values on different NMR spectrometers, or on different high-field MR scanners^18^,^19^. Such time consuming measurements provide information for the efficiency of the relaxation enhancement by contrast agents at discrete values of the magnetic field only, and cannot be routinely performed for the design of new contrast agents in a chemical laboratory having a single high-field NMR magnet.

An alternative approach to the use of pulsed magnetic fields for NMRD studies, is the use of rapid sample translation between positions in the stray magnetic field produced by the NMR magnet^20^,^21^,^22^,^23^. This approach offers the possibility to reach a wider range of magnetic fields, which can cover the entire span between the field values at the center of the magnet and virtually a null magnetic field. The major disadvantage lies however in the significantly longer time that the sample requires in order to travel between the locations of pre-polarization, relaxation and detection which can be of the order of several hundreds of milliseconds. Rapid sample shuttling is achieved using pneumatic^21^,^24^,^25^, or mechanic technologies^20^,^22^,^23^. Mechanical technologies are much more versatile and precise and can be easily used with standard NMR hardware as we recently demonstrated in the area of bio-molecular high-resolution fast field cycling relaxometry^22^,^23^,^26^.

Here we use our rapid mechanical sample-shuttling device for performing *T*_1_ relaxation measurements covering an ultra-wide range of magnetic fields in a virtually continuous fashion^23^,^27^. We demonstrate the high reliability of the measurements by observing the equilibrium magnetization of newly designed mixed Gd-Eu vanadate nanoparticles at different magnetic fields and perform a field-dependent study of *T*_1_ relaxation on three different nanoparticle compositions and sizes. In contrast to Gd chelates, the particles present a maximum contrast performance for magnetic fields between 1 and 1.5 T. We use the Magnetic Particle (MP) model^28^ to fit the data and discuss the extracted parameters. These results display how using ultra-wide range and continuous field dependent measurements of relaxivity should allow scientists to better understand crucial factors for relaxation, such as the nanoparticle microstructural properties, thus opening the way toward a rational design of optimized contrast agents for high filed MRI.

## Materials and Methods

### High-field high-resolution relaxometer

**Figure 1:**
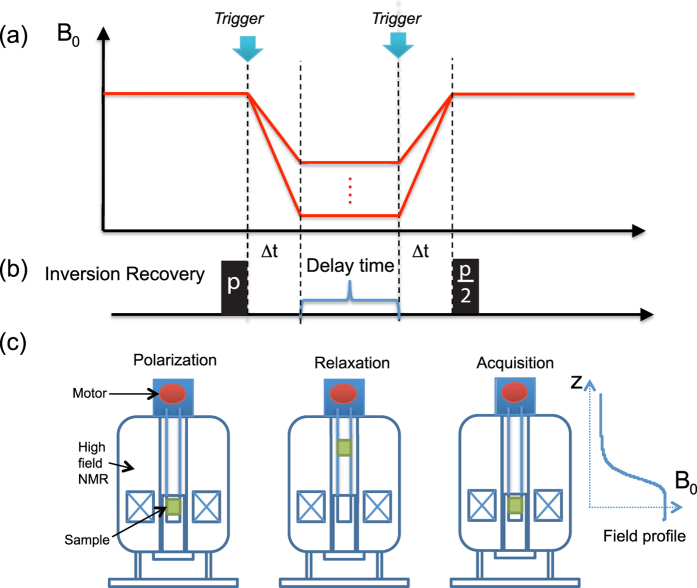
Rapid sample shuttling for field-dependent measurements of longitudinal relaxivity: The change of the magnetic field that the sample experiences, as a function time axis, is presented in (**a**). The inversion recovery pulse sequence, shown in (**b**), is synchronized with the sample shuttling in order to allow for evolution of the magnetization towards equilibrium in the presence of the low magnetic field, during the delay time. The sample position inside the superconducting NMR magnet during the experiments is shown in (**c**). The sample position is synchronized with the pulse program.

A high speed sample shuttling instrument, named field-cycler, based on the design of Chou *et al*.^23^ was installed to a 500 MHz wide-bore superconducting magnet equipped with a 5-mm broadband homebuilt radio-frequency probe-head. The sample can be shuttled from the center of the magnet (at 11.7 T) to the top of the magnet (at <0.01 T) in 80 ms. Lower values of the magnetic field can be easily achieved using a μ-metal cylindrical box around the rail of the shuttle^29^. The magnetic field profile along the center axis was measured precisely using a Senis 3MH3-20T Hall probe. The probe was mounted on the shuttle and a high-resolution profile was acquired prior to the experiments. The radio frequency saddle-coil of the homebuilt broadband probe-head was specially designed to accommodate a small sample volume of 5 mm in length and 5 mm in diameter, in order to minimize position-dependent relaxation in the presence of a strong stray field gradient. An option to improve this without restricting the sample volume would be the generation of multiple homogeneous sweet spots as proposed recently^30^, but was not performed in the present work. The magnetic field strength at a given shuttle position was determined from the stray field profile. A programmable motor controller, as depicted in Fig. 1, drives the sample shuttling device. The sample was polarized at the center of the magnet before being shuttled to the desired upper positions for spin relaxation at various lower magnetic field strengths. After a predefined but variable time period in the presence of the low magnetic field, the sample was shuttled back to the high field center for signal acquisition. In Fig. 1, Δt is the sample shuttling time, equaling the effective field switching time. This time is controlled precisely by the power applied to the motor and stays constant during the relaxivity experiments. The high speed shuttling instrument has been operated for 10 million cycles without damaging the NMR sample tube nor to the probe-head. Because of its design, it can be adapted virtually to any modern commercial high-field NMR spectrometer.

### Sample preparation

The synthesis protocols and material properties of the Gd/Eu nanoparticles were previously published^14^. These particles are obtained by precipitation at low temperature. Two different synthesis protocols yielded 30-nm (normal route) and 5-nm nanoparticles (citrate route). The normal route synthesis consists in mixing rare earth nitrates with sodium orthovanadate at room temperature. These ions precipitate and form rare earth vanadate nanoparticles. The citrate route synthesis differs from the normal route by the addition of a citrate complexing agent that limits the particle growth. In both cases, the obtained nanoparticles were purified by centrifugation and washings, or dialysis in order to eliminate residual reactants and counter ions.

The particles were characterized with dynamic light scattering, transmission electron microscopy (TEM), X-ray diffraction and zeta potential measurements^14^. During all experiments, the samples were suspended homogeneously in distilled water as a colloidal dispersion. All samples were sonicated immediately prior to the measurements to disperse transiently formed aggregates. Bare 30-nm particles present a low surface charge (8.6 mV for Gd_0.6_Eu_0.4_VO_4_ particles) and tended to partially precipitate during the acceleration periods transporting the sample between the different magnetic field locations. We therefore used SiO_2_-coated nanoparticles obtained by silicate condensation at room temperature in water^31^. Silica-coated nanoparticles present a higher surface charge (−31 mV for Gd_0.6_Eu_0.4_VO_4_ particles) and thus show higher colloidal stability and do not precipitate in the experimental conditions. Note that the presence of a silica coating changes the relaxivity of the nanoparticles only slightly^14^. This is not surprising given the amorphous nature of the silica coating^31^ which allows the penetration of small molecules^14^,^32^. 5-nm nanoparticles obtained with the citrate route have a surface charge of −13 mV due to the presence of citrate ions at the particle surface and did not require any further coating.

The *R*_1_ of background water was measured in the suspension buffers after removal of the nanoparticles by centrifugation and filtration. We centrifuged the solutions containing the nanoparticles during 10 minutes at 10000 g. The supernatant was then filtered by centrifugation, 5 min at 5000 g, through a 10 kDa (equivalent to 10 kg/mol) molecular weight cut-off membrane to filter out any remaining small particles and ensure that the filtrate only contained solvent molecules.

### Concentration determination

All sample concentrations were set to 0.1 mM in vanadate ions. Relatively low concentrations were chosen so that the relaxation during the 80-ms shuttling time remains negligible. The vanadate concentration was determined after nanoparticle dissolution with a mix of hydrochloric acid and hydrogen peroxide that causes the formation of orange-brown colored vanadium complexes. The detection of their specific absorption peaks at 405 nm and at 460 nm leads to the precise colorimetric quantification of vanadate ions in the colloids^33^. Assuming stoichiometric composition, the Gd^3+^-ion concentration can be determined from the vanadate ion concentration by multiplying with 0.6 for the Eu-doped particles and with 1 for GdVO_4_ particles. To calculate the nanoparticle concentration from the vanadate ion concentration, we used the nanoparticle volume and the volume of the Gd_1−x_Eu_x_VO_4_ unit cell (V_u.c._ = 330 × 10^6^ pm^3^ for GdVO_4_) to determine the number of unit cells and, subsequently, the number of vanadate ions per nanoparticle for each type of nanoparticles, as explained in Casanova, D. *et al*. in 2006 34. Note that each unit cell contains 4 vanadate ions. To determine the nanoparticle volume, we used the nanoparticle dimensions as obtained from TEM measurements and assumed that the third dimension is equal to the smaller of the two axis dimensions (*i.e.* that the particles are prolate spheroids). In the case of the larger particles, their two-dimensional projection is an ellipse with the two major axes having lengths of 13.1 ± 1.1 and 26.6 ± 4.8 nm (mean ± standard deviation); in the case of the smaller ones, their two-dimensional projection is a circle with diameter 5 ± 0.2 nm^14^. We thus obtained from the measured 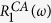 values in s^−1^, the relaxivity values 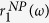 in mM^−1^.s^−1^, normalized to the nanoparticle concentration for comparison with the Magnetic-Particle model described below.

### Data Validation

**Figure 2:**
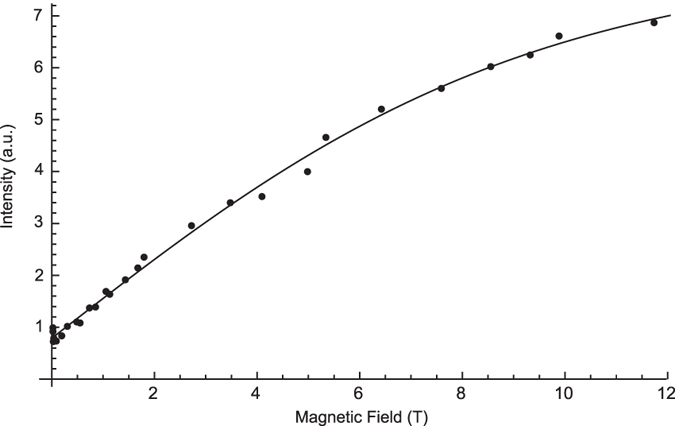
Profile of the equilibrium magnetization as a function of the equilibrium magnetic field as extracted from the field-dependent relaxivity measurements of 5-nm Gd_0.6_Eu_0.4_VO_4_. A long delay of 20 s during the inversion recovery pulse sequence was applied. This curve follows closely the Langevin equation (solid line).

**Figure 3:**
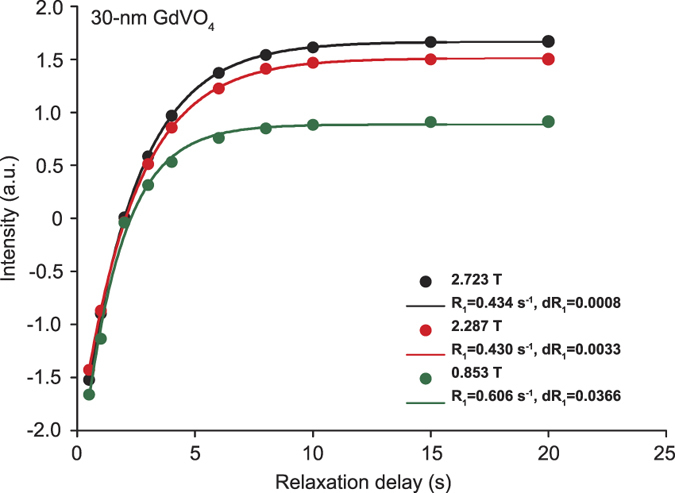
Inversion recovery curves collected at different magnetic fields for the 30-nm GdVO_4_ nanoparticles. Measured data points are shown as dots and the fitted curves are presented with solid lines. Three different magnetic fields were used: 2.723 T (black), 2.287 T (red), and 0.853 T (green).

To demonstrate the reliability of the field-cycler on the application of relaxivity measurement, the peak intensities after a long relaxation delay time, 20 s, at different fields were extracted from the relaxation rate R_1_ measurements. The residual equilibrium magnetization was measured by applying a *π*/2 pulse immediately after the sample was shuttled back to the center of the magnet. The experimental field-dependent residual equilibrium magnetization is shown in Figs 2 and 3. According to the Langevin function^35^:


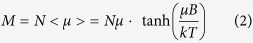


where *B* is the magnetic field, *N* is the total number of spins, T is the temperature, *μ* is the magnetic moment, *M* is the magnetization, and *k* is the Boltzmann constant. In the high temperature limit, 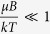, the correlation between magnetic moment and magnetic field is considered to be a positive linear function, which is the well-known Curie’s law^35^. Our experiments were performed at a constant room temperature of 300 K. The measured field range covers from 11.7 T to the fringe field of 0.01 T, where 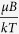 is not in the limit case of 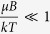, over the full range of magnetic fields. The field-dependent profiles of our experimental measurements were found to be in good agreement with hyperbolic tangent function, as shown for an example curve in Fig. 2, for a sample containing 5-nm Gd_0.6_Eu_0.4_VO_4_ nanoparticles (the correlation coefficient of the fit was higher than 0.97). This result demonstrated the high accuracy and high reliability of our sample shuttling device and therefore of the relaxivity measurements. Figure 3 demonstrates the inversion recovery curves for 30 nm GdVO_4_ nanoparticles at low fields.

## Results and Discussion

### Measurements of NMRD curves

**Figure 4:**
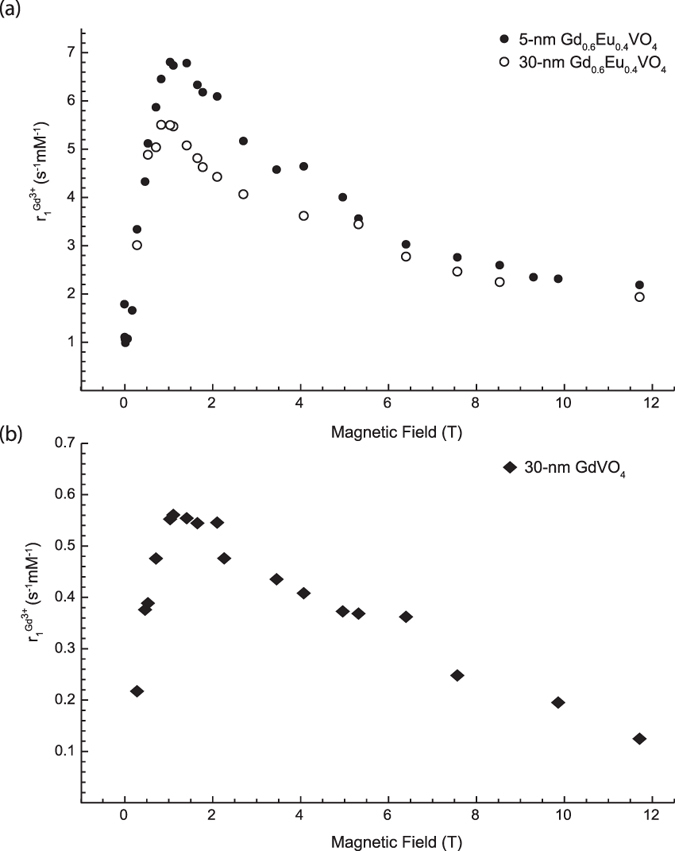
The relaxivity profiles of 5-nm and 30-nm gadolinium vanadate nanoparticles with and without Eu-ion doping are presented as a function of the magnetic field. The relaxivity shows the relaxation enhancement divided by the concentration of Gd ions. In (**a**), relaxivity values of 5-nm and 30-nm Gd_0.6_Eu_0.4_VO_4_ particles are displayed with solid and open circles, respectively; the values of 30-nm GdVO_4_ particles are displayed with solid diamonds in (**b**). These data represent the efficiency of relaxation enhancement based on the Gd concentration. This means that the 5-nm Gd_0.6_Eu_0.4_VO_4_ colloid provides the most efficient relaxation enhancement for a given Gd^3+^ concentration. For a fixed desired relaxation enhancement, it would be the contrast agent requiring the administration of the lowest Gd-ion concentration.

The contrast agents studied here were vanadate nanoparticles having two different sizes, citrate-stabilized 5-nm and silicate-stabilized 30-nm particles, and two different compositions, GdVO_4_ and Gd_0.6_Eu_0.4_VO_4_. These nanoparticles were shown to be multifunctional combining the functionalities of luminescent labels, oxidant sensors, and MRI contrast agents^14^. In particular, the administration of 5-nm and 30-nm Gd_0.6_Eu_0.4_VO_4_ nanoparticles into a mouse lead to considerable contrast enhancement in different organs in *in vivo* MRI images acquired at 200 MHz (4.7 T)^14^. Our previous work included the measurement of NMRD profiles for frequencies up to 20 MHz (0.469 T). These data indicated an increase in relaxivity above 10 MHz but did not encompass high enough frequencies to be able to determine if there is a maximum relaxivity and the corresponding magnetic field^14^. The data obtained here (Fig. 4) confirm these previous data and enable the determination of the full NMRD profile up to magnetic fields of 11.7 T.

All NMRD profiles were recorded by applying an inversion recovery pulse sequence. First the sample was left to relax for 15 s at the center of the magnet. After an inversion π pulse (22.5 μs), the sample was rapidly sent to the lower magnetic field and left there for the duration of the relaxation delay. Subsequently, the sample was shuttled back to the initial position where transverse magnetization was detected following a hard π/2 pulse (11.25 μs). 8 scans were recorded for each value of the magnetic field. 25 values of the magnetic field were explored for a total acquisition time of 21 hours per sample.

The three main requirements of this sample shuttling NMRD approach are the following: First, the samples should not precipitate under acceleration during the transfer between the different magnetic field locations. This is the case for most stable colloidal solutions. In our case, nanoparticles precipitate for accelerations on the order of 5000 g for the 30-nm nanoparticles and >10 000 g for the 5-nm particles, with spinning durations of several minutes. In our measurement, the samples experience the shuttling acceleration, which is lower than 20 g and much lower than the acceleration required to precipitate the nanoparticles. Second, the relaxation times should be long compared to the shuttling time of 80 ms. This is easily achieved by lowering the concentration of the contrast agent, as mentioned above. Third, the particles should not precipitate in the presence of large field gradients. This was not the case for the paramagnetic nanoparticles described here.

There are two possible ways to present *r*_1_(*ω*): either normalized to the Gd^3+^ concentration^14^,^15^ for general medical purposes, or normalized to the nanoparticle concentration in order to investigate the molecular and hydrodynamic effects on the relaxivity. The normalization to the Gd^3+^-ion concentration is more relevant if we are interested in determining whether small (5 nm) or large (30 nm) nanoparticles are more efficient as contrast agents for a fixed Gd^3+^-ion concentration. For understanding the hydrodynamic mechanism on the nanoparticle scale, the comparison and fitting with the Magnetic-Particle (MP) model described below normalization by the nanoparticle concentration is essential and is designated in the following discussion as 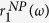. The obtained NMRD profiles of colloidal nanoparticle solutions of two different sizes and two compositions are shown in Fig. 4, where the 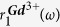 values are normalized by the concentration of Gd^3+^-ions. All three samples were found to have a maximum r_1_ value around 1–1.5 T in an asymmetric hump of the NMRD curves. The maximal values for the three NMRD profiles of Fig. 4 are:
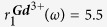
 s^−1^mM^−1^ for the 30-nm Gd_0.6_Eu_0.4_VO_4_ colloid, 6.8 s^−1^mM^−1^ for 5-nm Gd_0.6_Eu_0.4_VO_4_ particles, and 0.56 s^−1^mM^−1^ for 30-nm GdVO_4_ particles. These measurements normalized to the Gd^3+^-ion concentration reveal a relaxation enhancement maximum in the following order: 5-nm Gd_0.6_Eu_0.4_VO_4_ > 30-nm Gd_0.6_Eu_0.4_VO_4_ > 30-nm GdVO_4_. Interestingly, the Eu-doped vanadate nanoparticles were found to have higher maximal *r*_1_ than the non-doped ones. This effect was also observed in our previous work in the low-field range^14^. We attribute it, at least partially, to the contribution of the Eu^3+^-ion magnetic moment due to non-zero total angular momentum J at room temperature, known as the Van Vleck paramagnetism of europium^36^,^37^. Indeed, the ground state of the Eu^3+^-ion has a zero magnetic moment. However, the excited states of Eu ions possess non-zero magnetic moment and are located close to the ground state. They are therefore partially occupied at room temperature. This magnetic contribution is not taken into account because we normalize with the Gd^3+^ concentration resulting in seemingly higher relaxivity values for the Eu^3+^-doped colloids. Another possible contribution to the enhancement of relaxation properties in Eu-doped particles may result from structural differences. Doping with different ions could potentially modify the crystallinity of the particles and might yield a greater access to water molecules.

For a same chemical composition, the dispersion curves also indicate that the smaller nanoparticles are more efficient relaxing agents. 5-nm Gd_0.6_Eu_0.4_VO_4_ particles displayed a distinct NMRD profile with significantly higher maximal relaxivity than 30-nm Gd_0.6_Eu_0.4_VO_4_ particles (Fig. 4). This effect is consistent with a higher surface-to-volume ratio for smaller particles, which implies that water molecules have direct access to a larger fraction of Gd^3+^ ions in smaller particles. It is also consistent with the correlation between the reduction of nanoparticle size and longitudinal relaxivity enhancement observed for MnO_2_ particles^38^. However, Gd_0.6_Eu_0.4_VO_4_ relaxivities are almost size-independent for field values higher than 8 T or lower than 0.55 T (*r*_1_(9.9 T;5-nm Gd_0.6_Eu_0.4_VO_4_) = 2.321 s^−1.^mM^−1^ and *r*_1_(9.9 T; 30–nm Gd_0.6_Eu_0.4_VO_4_) = 2.254 mM^−1^s^−1^).

Moreover, our previous work showed that the presence of a silicate layer on the 30-nm particles induces only a small change in their relaxivity. Indeed, we measured at 20 MHz a 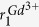 value of 3.47 and 3.03 mM^−1^.s^−1^ for bare and silicate-coated Gd_0.6_Eu_0.4_VO_4_ 30-nm particles, respectively, corresponding to 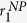 values of 67 300 and 58 800 mM^−1^.s^−1^, respectively. Note in particular that the magnetic field value corresponding to 20 MHz (0.47 T) lies in the range of the relaxivity increase due to the onset of the peak reaching a maximum at 1–1.5 T (see Fig. 4). This small effect of the silicate coating cannot explain the large 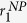 difference observed between 5-nm and 30-nm nanoparticles, i. e. about 2 200 and 82 000 mM^−1^.s^−1^, respectively, as discussed in the following. The small influence of the silicate layer is not surprising given that i) its thickness is small according to ref.^31^, and ii) it is only partially condensed and permeable to small molecules. The latter point was confirmed by our previous observation that H_2_O_2_ molecules can penetrate the silicate layer and oxidize 30-nm reduced Y_0.6_Eu_0.4_VO_4_ particles^32^.

Thus, both the chemical compositions and the size of the nanoparticles contribute to the relaxivity properties. Moreover, the nanoparticle microstructure in terms of crystallinity, surface properties, and internal porosity should also influence the water accessibility to the inner part of the particles and consequently the relaxivity properties. To gain further insight into these microstructure effects, we compare our results with the Magnetic-Particle relaxation model in the following section.

### Model Analysis

Amongst the relaxation models for contrast agents, the most notable and classic one is the “Solomon-Bloembergen-Morgan model” (or SBM model)^39^,^40^. This model describes far field dipolar and contact interactions between protons and contrast agents. The SBM model is suitable for analyzing the relaxation enhancement induced by paramagnetic molecules, such as Gd-chelates. The extension of its dipolar component to nanoparticles^14^,^38^,^41^,^42^,^43^,^44^,^45^,^46^, the so-called magnetic particle (MP) model, has been developed recently and applied to crystalline Fe_2_O_3_ nanoparticles^28^. In solutions of magnetic nanoparticles, the dipole interactions between the nuclear spin of the water protons and the electronic magnetic moment of the nanoparticle is the dominant mechanism for the nuclear spin relaxation of the protons^28^. The relaxation mechanisms of magnetic nanoparticles were modeled by considering two contributions due (i) to the thermal average value of the Curie spin of the nanoparticles 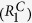 and (ii) to the fluctuations of the nanoparticle electronic magnetic moment around its thermal average value 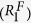:


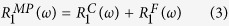


Given the size of the nanoparticles, the rotational diffusion of the nanoparticles is too slow to affect the relaxation and can be neglected^47^,^48^. The particle dynamics are thus completely described by the knowledge of the translational diffusion, *i.e.* the relative motion of the particle and of surrounding water molecules, characterized by the diffusion correlation time *τ*_*trans*_. Since the diffusion of nanoparticles is several orders of magnitude slower than the diffusion of water, *τ*_*trans*_ depends only on the effective diffusive properties of water molecules at the surface of the nanoparticles, mostly determined by the nanoparticle surface chemistry and microstructure. Hence, the spectral density function was given as^48^.


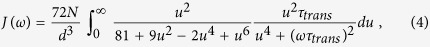


where *N* is the number of nanoparticles per unit volume, *d* is the closest distance between the nanoparticle electronic magnetic moment and the proton spin.

To describe the Curie spin relaxation, the classical spin factor in the SBM model is replaced by the Curie spin 
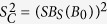
, where *B*_*S*_(*B*_0_) is the Brillouin function. Consequently, the Curie relaxation rate was written as:


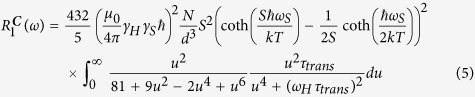


The relaxation due to the fluctuations of the nanoparticle electronic magnetic moment was written as:


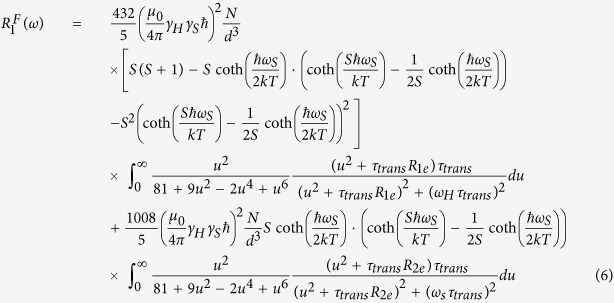


Here, *R*_1*e*_ and *R*_2*e*_ represent electron spin-lattice relaxation rates, which are assumed to be field-independent^28^ and have a typical magnitude of 1–10 ns^−1^ 28,49. Constants in Equation (5) and (6) are: permeability, *μ*_0_; Boltzmann constant, *k*; gyromagnetic ratio of proton and paramagnetic spin, *γ*_*H*_ and *γ*_*S*_, respectively, and the temperature T =  300 K in our measurements. The control variables here are Larmor frequencies of the proton and of the paramagnetic spin, *ω*_*H*_ and *ω*_*S*_, respectively.

**Figure 5:**
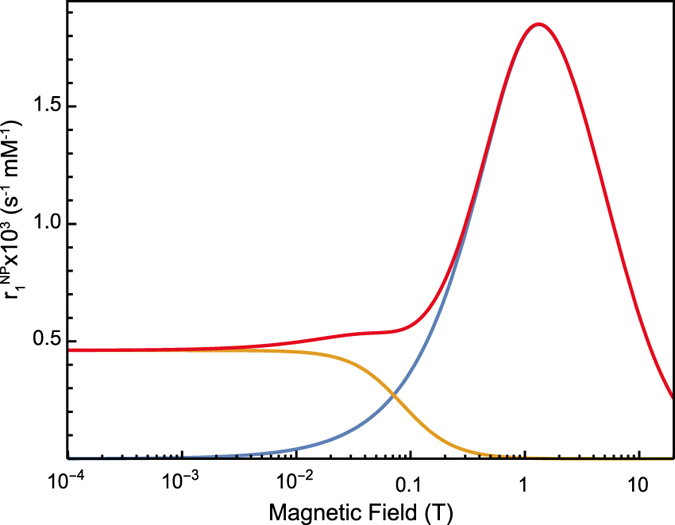
Simulation curves of 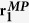 and its contributing functions, 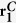 and 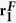, in a log plot for the magnetic-field axis. The parameter values we used were: *S* *=* 4000, *d* = 2.2 *nm, τ* = 14 *ns, R*_1*e*_ = 10 *ns*^−1^, *R*_2*e*_ = 2 *ns*^−1^. 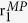 is shown in red, 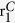 is shown in blue and displays a high-field dependency in the field range above 0.1 T. The field dependency of 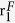 (orange line) occurs in the field range of 0.01 T to 1 T. For magnetic fields above 1 T, 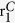 becomes the dominant contribution for the nonlinear curve of 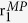.

This model provides a physical picture of hydrodynamic (*τ*_*trans*_, *d*) factors controlling water proton spin relaxation mechanisms due to paramagnetic nanoparticles. The field dependency of 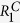 and 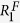 and *R*_1_ functions are presented in Fig. 5. The hydrodynamic parameters (*i. e.* the translational diffusion) impact mainly on the 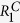 function, which has high field dependency for fields higher than 1 T. The relaxation function 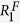 dominated by the electronic spin fluctuations shows less field dependency in the field range higher than 1 T and is much weaker than 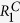 above 0.1 T.

This model has been successfully applied at different temperatures only within the low-field range from 10 kHz to 20 MHz for Fe_2_O_3_ nanoparticles^28^. This low field range could not reveal the nonlinear behavior of relaxivity caused by the hydrodynamic coupling that can induce an important relaxation enhancement at magnetic fields higher than 1 T.

### Discussion of the fitting parameters

Our experimental data were fitted to the MP model using the equations presented above and programing in Mathematica. The fitting parameters were: the typical distance between the paramagnetic particles and the water proton, *d*; the translational correlation time, *τ*_*trans*_; the spin number, S; and the electron spin-lattice and spin-spin relaxation rates, *R*_1*e*_ and *R*_2*e*_.

The fitting procedure was the following: (i) in order to find initial values for the fitting parameters suitable for further optimization, we fit the experimental data only with 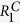 from 1.5 T to 11.75 T, (ii) the optimal parameters were determined by fitting the relaxivity data with the full function of R_1_^MP^.

We observed large tolerances with respect to the value of the electron spin relaxation rates R_1e_ and R_2e_ (0.1 to 10 ns^−1^), which indicates that any values within the ns range are compatible with our data. This is due to the fact that the contribution of the function 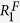 related to the fluctuations of the electronic magnetic moment is small compared to the term 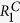 and can be neglected for the field range we are interested in. Figure 5 indeed shows that the 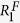 function contribution becomes important only for low fields below 0.1 T. The major contribution is thus due to the component R_1_^C^, dependent on hydrodynamic parameters. We therefore used only the R_1_^C^ component to fit the data.

**Figure 6:**
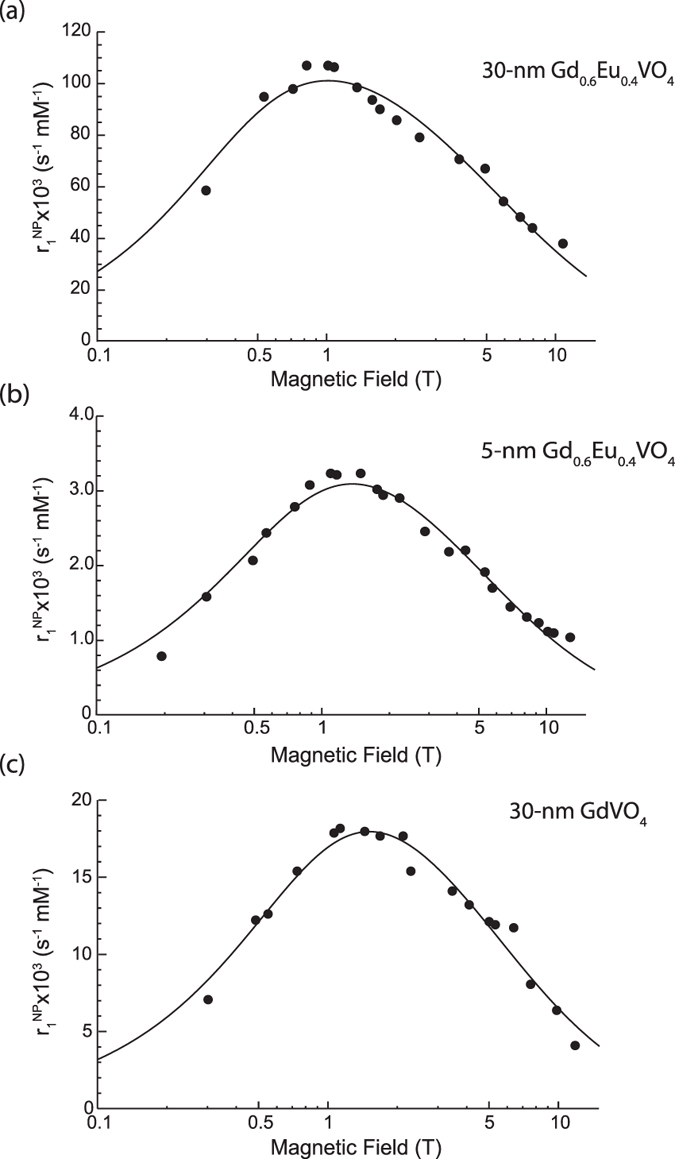
Relaxivity profiles and fits (solid lines) based on the MP model. Experimental measurements of relaxivities on different particle solutions are presented in dots. The relaxation enhancement values have been divided with the nanoparticle concentration. (**a**) 30-nm Gd_0.6_Eu_0.4_VO_4_, (**b**) 5-nm Gd_0.6_Eu_0.4_VO_4_, and (**c**) 30-nm GdVO_4_ nanoparticles.

Figure 6 shows the overall fitting of NMRD profiles for the three different nanoparticles. The determined parameters *d, τ, S* are presented in Table 1 The relaxivity values were calculated per nanoparticle, *i.e.* the relaxation rate changes due to the nanoparticles were divided with the nanoparticle concentration [see Eq. (1)].

Let us first examine the parameters obtained for the 5-nm Gd_0.6_Eu_0.4_VO_4_ nanoparticles. (Table 1). The distance between the paramagnetic nanoparticle and the water proton (*d* = 1.9 ± 0.2 nm) is found to be close to the value expected from the nanoparticle radius, 2.5 nm. This finding is similar to the results of Kruk *et al*.^28^, who found distances *d* to be consistent with the nanoparticle radii. The solvent diffusion coefficient can be approximated by the square of the distance between water protons and the paramagnetic particle divided by the translational correlation time, *D *=* d*^2^/*τ*. We find 0.24* *×* *10^−9^* m*^2^/*s* which is lower than the water self-diffusion coefficient (2.2* *×* *10^−9^* m*^2^/*s*)^50^. This lower diffusion coefficient may be due to the presence of a solvation shell around the nanoparticles^51^.

Surprisingly, however, the parameter values found for the 30-nm Gd_0.6_Eu_0.4_VO_4_ and GdVO_4_ nanoparticles and, in particular, the particle-water proton distance *d*, do not correspond to the nanoparticle radius. Although the *d* values found are similar for Eu-doped and undoped 30-nm particles (0.80 ± 0.06 and 0.89 ± 0.07 nm, respectively), they are much smaller than the particle radius and even smaller than the distance value obtained for the smaller 5-nm particles. Moreover, the diffusion coefficients are also found to be similar for Eu-doped and undoped 30-nm particles (0.07 and 0.06* *×* *10^−9^* m*^2^/*s*, respectively) but are much smaller than the diffusion coefficient obtained for the 5-nm Gd_0.6_Eu_0.4_VO_4_ colloid.

These striking results for the distance *d* and the diffusion coefficient *D* can possibly be explained by taking into account the 30-nm particle microstructure. Contrast fluctuations in TEM images indeed evidence the inhomogeneous microstructure of the particles^14^. Gas adsorption measurements on 30-nm Y_0.6_Eu_0.4_VO_4_ particles synthesized with the same protocol yielded a specific surface 20 times higher than the specific surface expected from the nanoparticle geometric size^52^, so that the particles are expected to present a significant porosity and a rugged surface texture as a result of their formation process^53^. In contrast, structural investigations of the 5-nm particles support a monocrystalline (dense) structure^14^.

The presence of significant internal porosity, or a highly rugged surface of the 30-nm particles may explain both of our findings: the small nanoparticle-water proton distance and the low diffusion coefficient. The magnetic particle model assumes a perfectly crystalline structure. However, the deviations from crystallinity present in 30-nm particles may lead to an effective diffusion coefficient affected by the altered motion of water molecules around or inside the particles determined by their surface roughness and/or internal porosity.

Concerning the spin number, one may expect the 30-nm particles to be characterized by a larger spin number than the 5-nm particles because of the larger number of Gd^3+^ ions that they contain. However, given the size of 30-nm particles, and despite their microstructure, it is highly probable that a large number of Gd^3+^ ions are located too far away from the water molecules to contribute to the spin number involved in the dipole-dipole interaction with the water protons. Indeed, as mentioned above, these nanoparticles have some porosity, which allows to some extent water molecule penetration to a typical distance lower than the particle size, via a network of interconnected free spaces with a highly reduced effective diffusion coefficient. It is however probable that water molecules do not penetrate in the totality of the particle or, even if they do, they cannot be easily exchanged. Thus, only a subset of Gd^3+^ ions, possibly close the surface of the particles or to the surface of crystallites inside the nanoparticles, contributes to the interaction with protons. The total effective spin number obtained from the fits would thus result only from the contribution of these *surface* Gd^3+^ ions leading to spin number values lower than those expected if every ion was interacting with water protons (Table 1).

Fitting the NMRD profiles of nanoparticles of different sizes and compositions with the Magnetic Particle model showed that, for fields above 0.1 T, the contribution of nanoparticle magnetic moment fluctuations is negligible and that the relaxivity profile and its maximum value are dominated by hydrodynamic effects. The MP model considers monocrystalline particles. However, our data demonstrate that the internal nanoparticle microstructure may have a crucial influence on the hydrodynamic factors governing the nanoparticle relaxation enhancement properties, namely the water diffusion coefficient and the nanoparticle-water proton distance. Translational diffusion and water accessibility to the paramagnetic ions inside the nanoparticles due to varying nanoparticle microstructures require further investigations with relaxivity experiments, ideally in systems where the microstructure can be controlled. A better understanding of the hydrodynamics involved should lead to the establishment of appropriate relaxation models.

## Conclusions

We have successfully applied a novel approach to measure contrast agent relaxivities over a continuous and large range of magnetic field strengths going from potentially zero magnetic field, up to the center field of a commercial superconducting system, using our rapid sample shuttling device (the “field-cycler”). Our measurements showed a good robustness and allowed us to measure Gd and mixed Gd/Eu vanadate nanoparticle relaxivities for two different particle sizes. The Gd nanoparticles were found to significantly enhance proton relaxation (relaxivity r_1_ > 4 s^−1^mM^−1^) in a broad range of magnetic fields. The highest relaxivity occurred at a magnetic field of 1–1.5 T, which is in the range of the typical field used for clinical MRI. Comparing the r_1_ curves for these nanoparticles and fitting them with the magnetic-particle model, we demonstrated that the relaxivity efficiency correlates with both size and composition and that the relaxation enhancement was related to the water-accessible area as reflected by the size and porosity of particles. These results show that a great number of relaxation data could be routinely obtained on any commercial high-field NMR spectrometer. We can thus put them to the test and possibly improve on existing relaxation models. Our results obtained on Gd-Eu vanadate based nanoparticles are a striking illustration of how macroscopic properties such as the contrast in MRI can be controlled by the microstructure of the nanoparticles used as contrast agents. In this context, our method could be a central tool for the further MRI contrast agent rational design and optimization.

## Additional Information

**How to cite this article:** Chou, C.-Y. *et al*. Ultra-wide range field-dependent measurements of the relaxivity of Gd_1-x_Eu_x_VO_4_ nanoparticle contrast agents using a mechanical sample-shuttling relaxometer. *Sci. Rep.* **7**, 44770; doi: 10.1038/srep44770 (2017).

**Publisher's note:** Springer Nature remains neutral with regard to jurisdictional claims in published maps and institutional affiliations.

## Figures and Tables

**Table 1:** Fitted parameters of the MP model using the function 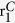 .

r_1_^C^	S (x1000)	*d* (nm)	*τ* (ns)	*D* = *d*^2^/*τ (m*^2^/*s* × 10^−9^)
*5-nm Gd*_*0.6*_*Eu*_*0.4*_*VO*_*4*_	4.0 ± 0.6	1.9 ± 0.2	15 ± 2	0.24 ± 0.05
*30-nm Gd*_*0.6*_*Eu*_*0.4*_*VO*_*4*_	6.8 ± 0.9	0.80 ± 0.06	10 ± 1	0.07 ± 0.01
*30-nm GdVO*_*4*_	3.4 ± 0.4	0.89 ± 0.07	13 ± 2	0.06 ± 0.01

The fits converged within 500 iterations. The parameter values are the best estimation solution with a 95% confidence level.
